# Synchronous pulmonary MALT lymphoma and squamous cell lung cancer: a case report

**DOI:** 10.1186/s12957-023-03069-8

**Published:** 2023-06-19

**Authors:** Zixin Guo, Liwen Hu, Qiongrong Chen, Junwei Hu, Jun Liu, Weidong Hu

**Affiliations:** 1 Department of Thoracic Surgery, Zhongnan Hospital of Wuhan University, Wuhan, 430071 People’s Republic of China; 2 Hubei Key Laboratory of Tumor Biological Behaviors & Hubei Cancer Clinical Study Center, Wuhan, 430071 People’s Republic of China; 3 Department of Pathology, Zhongnan Hospital of Wuhan University, Wuhan, 430071 People’s Republic of China

**Keywords:** MALT, Lung squamous cell carcinoma, Prognosis, Pathology

## Abstract

Pulmonary B-cell lymphoma in the extranodal marginal zone of mucosa-associated lymphoid tissue (MALT), a rare tumor originating from bronchial mucosa-associated lymphoid tissue, is the major histologic type of primary pulmonary lymphoma. Combined lung squamous cell carcinoma with pulmonary MALT lymphoma is rare. A 63-year-old male patient presented to the hospital because of a dry cough, and chest CT showed soft tissue density nodules in the upper lobe of the right lung, the boundary was visible lobulation and spiculation, and the middle lobe of the right lung showed patchy shadow, moderate enhancement, associated with bronchial traction. After a multidisciplinary diagnosis and treatment (MDT) discussion, surgical resection was done for the patient, and postoperative pathological results showed pulmonary MALT lymphoma combined with lung squamous carcinoma. For complex pulmonary multiple lesions, judgment needs to be made after MDT discussion, and timely intervention is required for lesions suspicious of malignancy. There are no uniform recommendations for the management of mixed tumors of the lung, and an individualized treatment plan needs to be developed based on the patient’s actual condition.

## Introduction

Mucosa‐associated lymphoid tissue (MALT) lymphoma is a low‐grade B‐cell lymphoma originating from extranodal MALT, which is the most common type of marginal zone B‐cell lymphoma among non‐Hodgkin lymphoma. MALT lymphoma often occurs in the stomach, lungs, salivary glands, thyroid, and orbital appendages [1]. Pulmonary MALT lymphoma, a rare tumor originating from bronchial mucosa-associated lymphoid tissue, is the major histologic type of primary pulmonary lymphoma, with an incidence of 0.5% of primary lung tumors [2]. Pulmonary MALT lymphoma is not characterized by clinical manifestations due to low incidence, slow progression, and extremely easy to be missed or misdiagnosed [3].

Lung squamous cell carcinoma accounts for approximately 25 ~ 30% of non-small cell lung cancer (NSCLC) and belongs to a common pathological type of lung cancer. Lung squamous cell carcinoma mostly originates from large bronchi and is more likely to cause obstructive emphysema or obstructive pneumonia [4]. It is very rare for both MALT lymphoma and squamous cell carcinoma of the lungs to simultaneously coexist in a patient [5].

This case report presents an elderly male patient with pulmonary MALT lymphoma and lung squamous cell carcinoma, including symptoms, diagnosis, and treatment of the disease.

## Case report

**Fig. 1:**
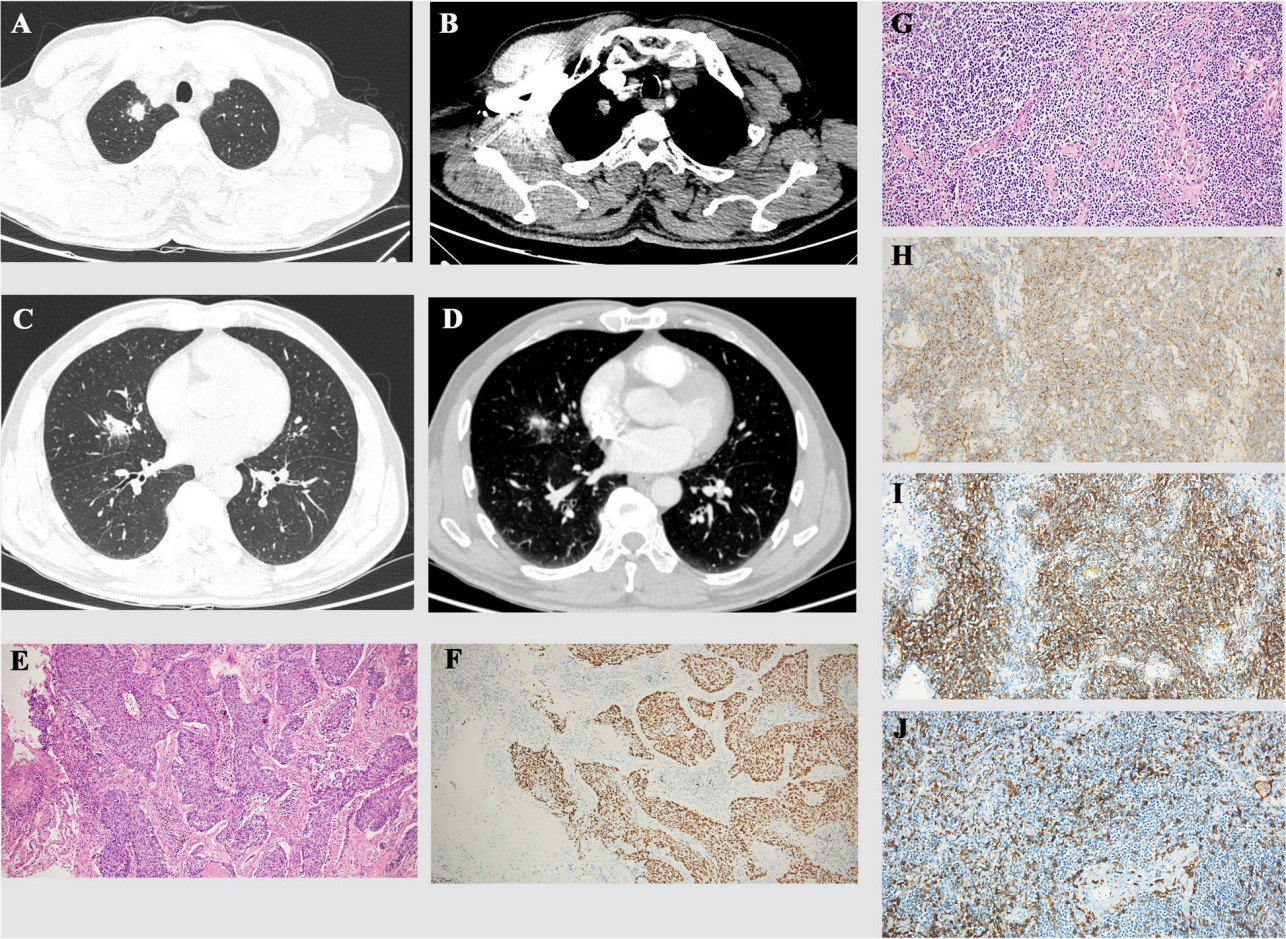
Pulmonary CT scan. A soft tissue density nodule was seen in the apical segment of the upper lobe of the right lung; the size was about 17 × 13 mm (**A** lung window, **B** arterial phase). Imaging features of MALT lymphoma in the lateral segment of the middle lobe of the right lung (**C** lung window, **D** arterial phase). Postoperative histopathological changes. **E** Squamous cell infiltrative growth (H&E, magnification × 100). **F** Cancer cells positive for P40 (P40, IHC, magnification × 100). **G** The morphology was more consistent with lymphoid cells infiltrating in sheets, with scant cytoplasm and deep nuclear staining (H&E, magnification × 200). **H** Cells CD20 diffusely (+) (CD20, IHC, magnification × 200). **I** CD21 staining revealed a network of follicular dendritic cells (CD21, IHC, magnification × 200). **J** CD3 negative (CD3, IHC, magnification × 200)

A 63-year-old male with a 40-pack-year smoking history presented with intermittent dry cough for 2 weeks, which failed to resolve with a dose of antibiotics. The patient did not have symptoms such as unintentional weight loss, fever, and night sweats, and a family history of tumors was denied. The pulmonary CT (computed tomography) showed a soft tissue density nodule in the apical segment of the upper lobe of the right lung, the size was about 17 × 13 mm, and the margins of which showed lobulation and spiculation. The above results indicated a high possibility of lung cancer (Fig. 1A, B). In addition to the lesions in the upper lobe of the right lung, there was a patchy shadow in the lateral segment of the middle lobe of the right lung, with moderate enhancement, which is related to bronchial traction (Fig. 1C, D). The patient subsequently had other imaging studies including brain magnetic resonance imaging (MRI), whole-body bone scan, and abdominal CT, which did not show metastasis. However, the patient did not undergo PET-CT for financial reasons. To better diagnose and treat patients, we conducted a discussion of multi-disciplinary treatment. The patient’s right middle lobe lesion was not suitable for percutaneous lung biopsy because the lesion was near the center, adjacent to the blood vessels, which are prone to danger and false negatives. At the same time, the patient was not suitable for tracheoscopic biopsy because the lesion was not in the trachea. Despite the absence of a clear pathological diagnosis, we considered these two nodules to have malignant potential and to be possible syndromic multiple primary lung cancer (MPLC), based on CT examination and multidisciplinary diagnosis and treatment (MDT) discussion. Considering the general status of the patient in combination, we planned to implement lesion resection surgery for the patient. Intraoperative exploration showed that the tumors were located in the upper lobe of the right lung and the middle lobe of the right lung, the tumor volume was small, no pleural invasion was seen, and intrathoracic lymph nodes did not show significantly enlarged fusion into masses. Wedge resection was first performed on the nodule in the upper lobe of the right lung, and the intraoperative frozen section pathological diagnosis was lung malignancy. Considering the location of the middle lobe lesion, the middle lobe lesion was not suitable for wedge resection, so the patient was treated with resection of the middle and upper lobes of the right lung. The postoperative pathological diagnosis revealed the following: a squamous cell carcinoma (nonkeratinizing type, size: 1.2 × 1 × 0.8 cm) in the upper lobe nodule of the right lung (Fig. 1E, F) and no carcinoma metastasis in any of the 12 lymph nodes sent, and the staging was T1bN0M0. The nodule in the middle lobe of the right lung was diagnosed as MALT lymphoma (Ann Arbor-Cotswolds stage system: IA stage), maximum infiltration diameter of about 0.6 cm. Immunohistochemistry (Fig. 1G–J) showed that it was CD20 (+), CD21 (+), CD23 (−), CD3 (−), CD5 (−), bcl-6 (−), CD10 (−), CD43 (−), CyclinD1 (−), kappa/lambda (about 1:1), and Ki-67 (proliferation index: about 10%). Monoclonal rearrangement of lymphocyte Ig gene (IGH: FR1-JH, IGH: DH-JH) was detected in the effective fragment of this sample. The patient completed a gastroenteroscopy after surgery, which excluded MALT lymphoma of gastrointestinal origin. According to the tumor stage of the patient as well as the individual wishes of the patient, no subsequent treatment was performed. We recommend that the patient have follow-up visits every 4–6 months.

## Discussion

Extranodal mucosa-associated lymphoid tissue marginal zone B-cell lymphoma (MALT) is a type of non-Hodgkin lymphoma that accounts for 5% of all non-Hodgkin lymphomas [6]. In 1983, Isaacson et al. [7] first reported two cases of MALT lymphoma in the gastrointestinal tract. With the development of MALT research, it is found that MALT lymphoma can also occur in the lung, salivary gland, thyroid, skin, and other tissues and organs, and the lung is the second organ prone to MALT lymphoma [8]. Primary pulmonary lymphoma (PPL) is very rare, accounting for 0.4% of all lymphomas and 3.6% of extranodal lymphomas. The most common type of PPL is MALT lymphoma of bronchial origin, which accounts for 70–90% of all PPLs [9]. The disease lacks specificity in clinical presentation, has diverse imaging features, and has a high rate of missed diagnoses and misdiagnoses [3]. At present, most scholars believe that the tumorigenesis of MALT lymphoma is initiated by long-term exposure to a variety of antigens (such as smoking, infection, autoimmune diseases), which is a defensive response [10, 11]. Further accumulation of cases of coexistence of these two malignancies and cytogenetic analysis is thus necessary to clarify this relationship.

Pulmonary MALT lymphomas occur mostly in men aged 60–70 years, and 38–50% of patients reported in the literature can be asymptomatic and detected only incidentally on physical examination [12, 13]. Clinical symptoms manifest as fever, cough, dyspnea, hemoptysis, chest pain, and other respiratory symptoms [14]. CT imaging of pulmonary MALT lymphomas is complex and of various forms, mostly along bronchovascular bundles or subpleurally and may show solitary or multiple nodules, clumps, or solid variants, often with intralesional air bronchograms, bronchiectasis, and ground glass density opacities around the lesion [15]. Hilum and mediastinal lymphadenopathy and pleural effusion are rare signs. In this patient, chest CT showed a single solid change, ground glass density shadow around the lesion, and bronchial traction, which were compatible with the imaging changes reported in the literature.

It is very rare for both MALT lymphoma and squamous cell carcinoma of the lungs to simultaneously coexist in a patient. The clinical manifestations of cough and dyspnea are common, and fatigue and weight loss are occasionally seen. The pulmonary CT shows bilateral lung consolidation, often accompanied by pulmonary nodules. Some patients have pleural effusion and pulmonary fibrosis. The diagnosis depends on pathological and immunohistochemical staining.

MPLC is defined as two or more primary malignant tumors that occur simultaneously or successively at the same or different sites in the lungs of the same individual and can be further divided into synchronous MPLC and metachronous MPLC by using the lesion occurrence interval of 6 months. There are still no clear treatment guidelines and protocols for MPLC, and there is a general consensus that surgical treatment is preferred for MPLC, and it should be combined with other treatment modalities for lesions that cannot be completely surgically removed [16]. MPLC has special biological characteristics, and after the publication of the TNM lung cancer staging system, each tumor lesion should be staged with separate T, N, and M, with the highest staging serving as the patient’s final stage [17]. This patient was a typical synchronous MPLC presenting with both primary lung squamous cell carcinoma and MALT.

Because the combination of pulmonary MALT lymphoma and lung squamous cell carcinoma is extremely rare, there are no guiding treatment recommendations for this type of mixed lung tumors at present. Single lesions of pulmonary MALT lymphoma can be treated surgically, and chemotherapy can also achieve better treatment outcomes if there is no indication for surgery. Most of the chemotherapy regimens were CHOP regimens (cyclophosphamide, doxorubicin, vincristine, and prednisone). The targeted therapy is mainly rituximab against CD20 antibody, which is combined with CHOP chemotherapy as a combination chemotherapy regimen, and the treatment effect is more desirable [18, 19]. The main treatment modalities for squamous cell carcinoma of the lung are surgery, radiotherapy, chemotherapy, and immunotherapy. Elderly patients often cannot receive effective complete treatment because of their physical condition and poor treatment tolerance. Lung squamous cell carcinoma has a poor prognosis and a short survival period. MALT lymphoma of the lung is an inert tumor that progresses slowly and does not experience extrapulmonary invasion in the short term [20, 21]. Priority should be given to the treatment of lung squamous cell carcinoma, while respecting the treatment wishes of patients and their families. The stage of lung squamous cell carcinoma in our patient was T1bN0M0, and surgery was the preferred option. Pulmonary MALT lymphoma is a solitary lesion and can be completely resected by surgery; while considering the willingness to treat and affordability of patients, no subsequent treatment was performed. For the treatment of mixed tumors of the lung, there is currently no uniform recommendation, and individualized treatment regimens need to be developed according to the actual conditions of patients.

## Conclusions

Our case, a patient with stage IA lung squamous cell carcinoma combined with stage I pulmonary MALT lymphoma, was diagnosed by preoperative imaging as well as postoperative pathology. This case suggests that multiple primary lung cancer requires multidisciplinary team management and partial lobectomy, and chemotherapy is an effective treatment for early cases.

## Data Availability

The data presented in this study are available on request from the corresponding author.

## Abbreviations

MALT: Mucosa‐associated lymphoid tissue
MDT: Multidisciplinary diagnosis and treatment
NSCLC: Non-small cell lung cancer
CT: Computed tomography
MRI: Magnetic resonance imaging
MPLC: Multiple primary lung cancer
PPL: Primary pulmonary lymphoma
